# Pictorial History of the Early Microscopic Study of Vagal Sensory Neurons

**DOI:** 10.1523/ENEURO.0143-26.2026

**Published:** 2026-08-11

**Authors:** Laurent Gautron

**Affiliations:** Department of Internal Medicine and Center for Hypothalamic Research, UT Southwestern Medical Center, Dallas, Texas 75390

**Keywords:** drawings, histology, history of science, interoception, microscopy, vagus nerve

## Abstract

Vagal sensory neurons play a central role in monitoring internal organ physiology and maintaining homeostasis. Although now recognized as key mediators of brain–body communication and promising therapeutic targets, their characterization emerged gradually over centuries of anatomical and microscopic investigation. This review provides a focused historical perspective on the study of vagal sensory neurons, emphasizing morphological and histological developments prior to the molecular era. Drawing on a collection of images from historical literature, this article traces the evolution of ideas and techniques, highlighting how early anatomical and histological studies established the foundation for modern investigations into vagal sensory function in health and disease. The review features the contributions of both well-known pioneers, including Adolph Hannover, Albert von Kölliker, Santiago Ramón y Cajal, and Fernando de Castro, as well as lesser-known investigators such as Adam Ploschko, Sergey Izhevesky, and Margaret Brown, whose work helped advance the field but has received comparatively little historical attention.

## Introduction: The Anatomical Era of the Vagus Nerve

Vagal sensory neurons are afferent neurons associated with the vagus nerve (cranial nerve X) that monitor the physiological state of internal organs and relay this information to the brain, thereby contributing to the automatic regulation of essential bodily functions. Their cell bodies reside in two ganglia: the superior (jugular) ganglion and the inferior (nodose) ganglion, the latter being the principal site of visceral sensory neurons. There is growing interest in therapeutic strategies aimed at modulating their activity (Ahmed et al., 2022), as well as in their involvement in human diseases. Excellent reviews on the anatomy and functions of the vagus nerve can be found somewhere else (Ottaviani and Macefield, 2022; Prescott and Liberles, 2022; Berthoud et al., 2025; Spencer et al., 2026).

This historical review provides a concise perspective on the microscopic study of vagal sensory neurons. I focus specifically on morphological and histological developments, rather than physiological or electrophysiological approaches, and restrict my scope to the premolecular era, prior to the widespread adoption of peptide- and DNA-based methodologies. The review is organized around major technical and conceptual advances, reflecting how methodological innovations shaped the field. It is not intended to be exhaustive but rather to highlight key contributors and milestones.

Before the advent of microscopy, vagal sensory neurons were not recognized as individual cells but were studied at the level of gross anatomy. The cranial nerves have been recognized since antiquity, with early descriptions attributed to Galen of Pergamon (129–216 CE; Porras-Gallo et al., 2019; Storey, 2022). However, the classification of peripheral nerves evolved gradually. The vagus nerve was not clearly distinguished as a separate entity from other cranial nerves until eighteenth-century anatomists, particularly Samuel Soemmerring (1755–1830) and Carolus Andersch (1732–1777), contributed to its differentiation (Flamm, 1967).

Notably, sensory functions of the vagus nerve were inferred early. Galen is reported to have recognized its sensory properties (Swanson, 2015), and by the Renaissance, several anatomists described it as conveying sensation from the viscera. For example, Ambroise Paré (1510–1590) referred to the vagus nerve as providing “sensation to the intestines and internal organs” (Paré, 1840).

**Figure 1.**
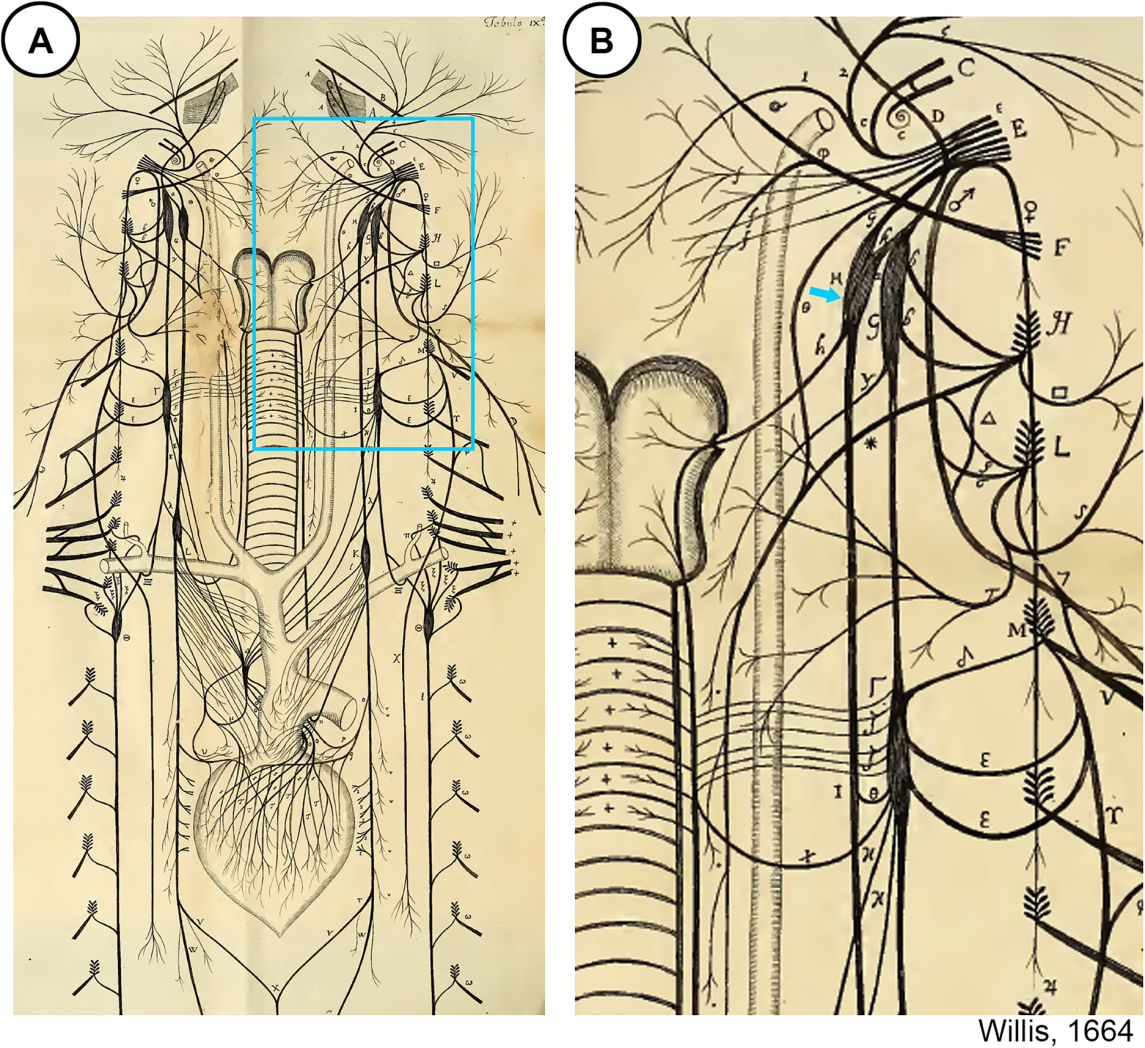
Drawings of the human peripheral nervous system from Willis published in 1664. The blue inset in ***A*** outlines the area shown in ***B*** after digital zoom-in. Willis labeled as the vagus nerve (“nervi paris vagi”) as “E”, a ganglionic plexus of the vagus (“plexus ganglioformis superior paris vagi”) as “H” and “h” as a branch to the muscle of the larynx (“ramus e plexu praedicto paris vagi in musculos laryngis”). Given that the laryngeal branch emerges from the nodose ganglion, one may deduce that the ganglion in H is the nodose (blue arrow). All images before 1931 are in the public domain and reproduced from Willis (1664) according to US Copyright Act (Title 17 of the United States Code).

Descriptions of the jugular and nodose ganglia date back to the sixteenth century. Gabriele Falloppio (1523–1562) described an “olive-shaped body” along the vagus nerve, likely corresponding to the nodose ganglion (Swanson, 2015). One of the earliest clear illustrations of vagal ganglia appears in the work of the English physician Thomas Willis (1621–1675; Willis, 1664). His depiction of a ganglion giving rise to a laryngeal branch likely corresponds to the nodose ganglion (Fig. 1*A*,*B*). Although no illustrations from Falloppio are known, later authors credited him with identifying ganglia (Ludwig, 1791). Christian Ludwig (1757–1823) credits him with a detailed description of the nodose ganglion as follows: “Fallopius, however, when discussing the vagus nerve—which, as is understood from his description of the nerve and enumeration of its branches, he calls the sixth nerve—maintains that certain small filaments arise from it which come together into an ‘olive-shaped body.’ This body is formed from a membrane that emerges from the skull and surrounds the vagus nerve. From this olive-shaped body, moreover, a fiber descends in front of the vertebrae of the neck, which, joined by certain filaments of the cervical nerves, forms additional ganglia beyond those mentioned above” (Ludwig, 1791).

**Figure 2.**
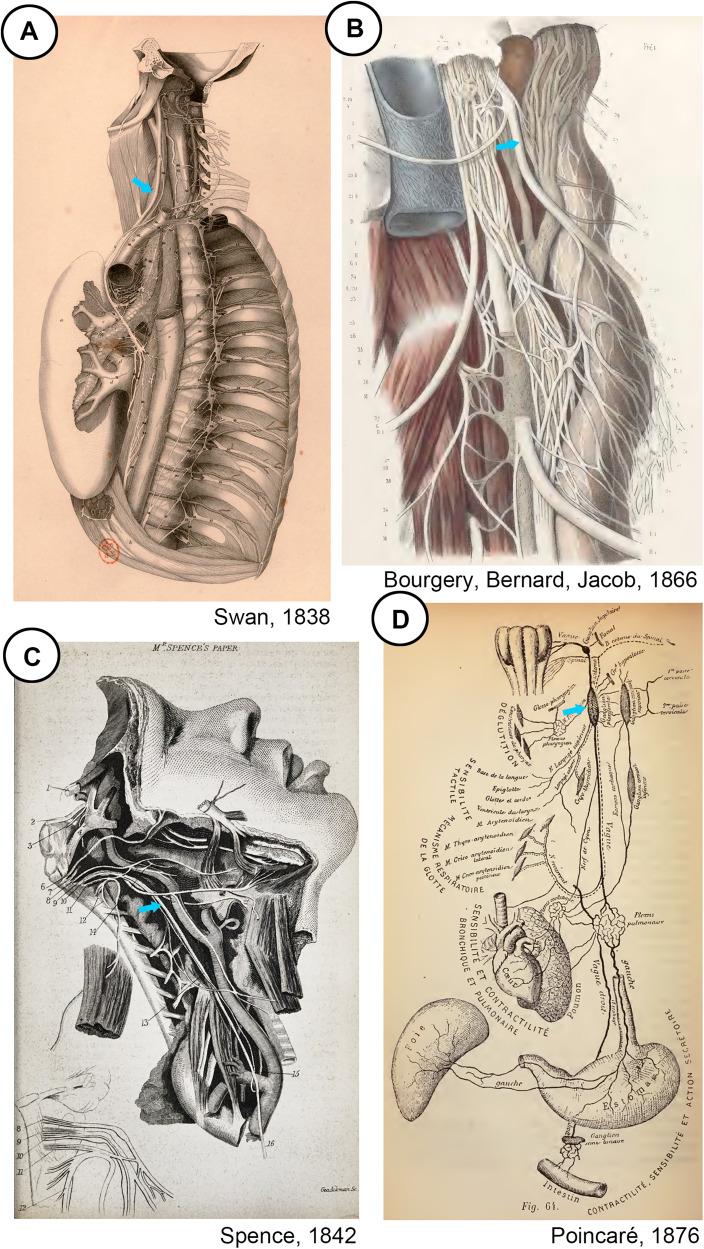
Representative illustrations of the vagus nerve and its associated ganglia in nineteenth-century anatomical treatises. ***A***, Drawing by the English anatomist Joseph Swan, reproduced from a commented French translation by Édouard Chassaignac featuring a delicate depiction of the vagus nerve (blue arrow). Image in the public domain reproduced from Swan (1838). ***B***, Detailed illustration of the cervical nerves from a work by Bourgery, Bernard, and Jacob, published in 1866. The colored figures by Jacob enhance the realism of the anatomical structures; the vagus nerve is indicated by a blue arrow. Image in the public domain reproduced from Bourgery (1866). ***C***, James Spence conducted notable anatomical studies of the human vagus nerve, including its sensory and motor components. The vagus nerve is indicated by a blue arrow. The bottom left corner includes a detailed depiction of the cranial ganglia and their interconnections. Image in the public domain reproduced from Spence (1842). ***D***, Émile Léon Poincaré authored a physiology text containing descriptions of the vagus nerve in 1876. This didactic drawing illustrates much of the nerve's trajectory across organs and labels its principal functions, largely consistent with modern understanding. Image before 1931 in the public domain reproduced from Poincare (1876) according to US Copyright Act (Title 17 of the United States Code).

By the mid-nineteenth century, detailed anatomical dissections had established the course, branching patterns, and visceral distribution of the vagus nerve. These studies, based on direct observation, often produced remarkably detailed and esthetically pleasing illustrations. Among numerous other examples, representative illustrations of the vagus nerve and its ganglia from that period of time are provided in Figure 2*A*–*D*. This anatomical tradition continued into the twentieth century, particularly in the context of vagotomy and, more recently, the development of vagus nerve stimulation.

With the rise of microscopy in the mid-nineteenth century, attention shifted from gross anatomy to cellular organization. While many of the central questions remained, such as the functional roles of the vagus nerve and its involvement in disease, the level of analysis changed fundamentally, setting the stage for modern neurobiology.

## Earliest Microscopic Observations of Vagal Sensory Neurons

**Figure 3.**
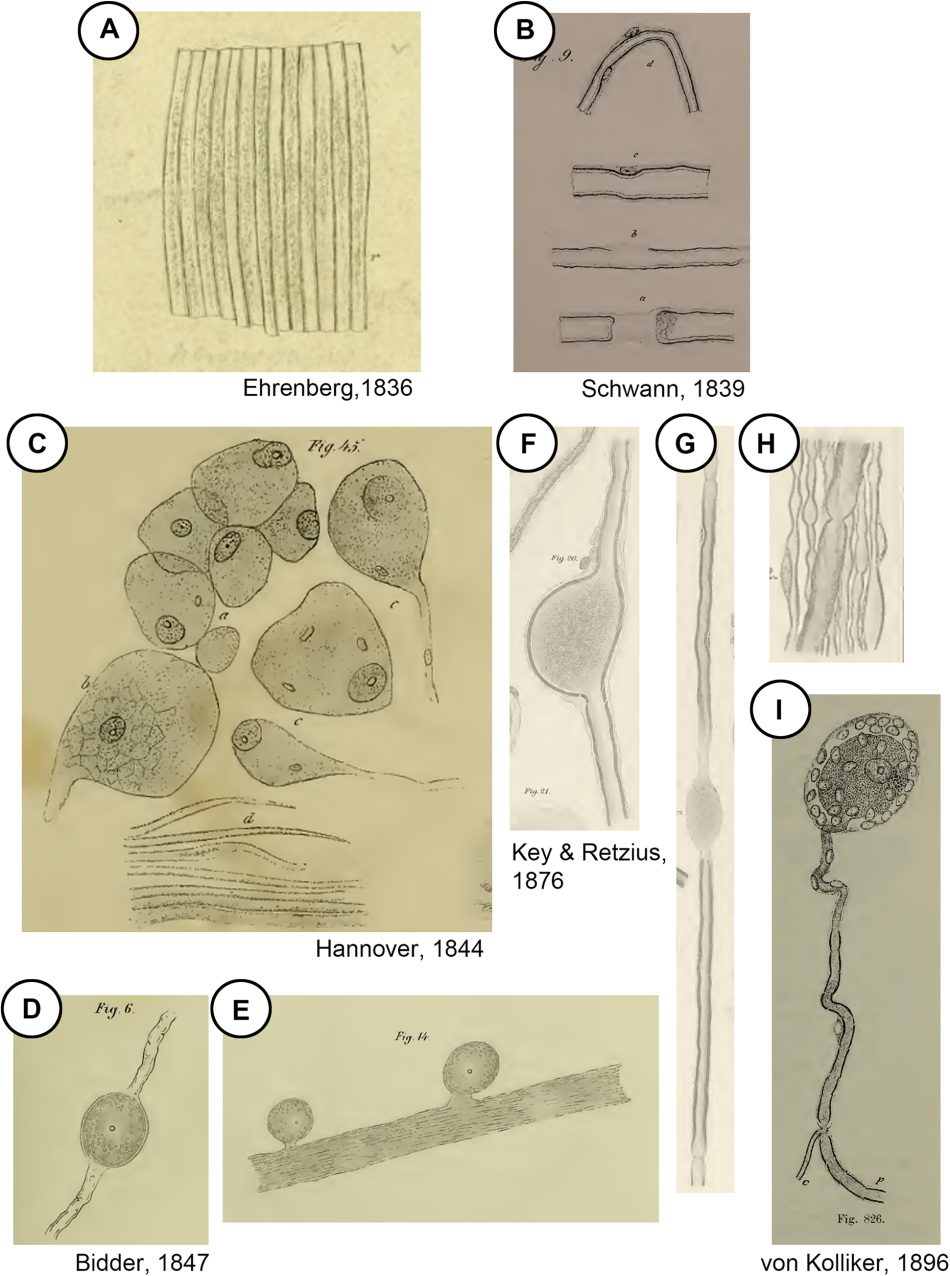
Early microscopic depictions of vagal sensory neurons and their fibers. ***A***, Christian Gottfried Ehrenberg made numerous microscopic observations of nerves and ganglia including the human vagus nerve. This could possibly be the first image of vagus nerve at microscopic level. Image in the public domain reproduced from Ehrenberg (1836). ***B***, Theodore Schwann described the calf vagus nerve. Image in the public domain reproduced from Schwann (1839). ***C***, Image obtained from the work of Adolph Hannover, showing clusters of ganglionic cells of the plexiform ganglion from flounder. Hannover noted variability in cell size, granular content, and attachment to nerve fibers. Image in the public domain reproduced from Hannover (1844). ***D***, Bidder studied ganglionic cells by microscopy, including those of the vagus nerve from pike. The axon is described as a “primitive tube.” Image in the public domain reproduced from Bidder (1847). ***E***, He also described ganglion cells arranged in a “berry-like” manner along the vagus nerve from a calf. Image in the public domain reproduced from Bidder (1847). ***F***, ***G***, Key and Retzius provided detailed drawings of pike preparations treated with osmium tetroxide, showing ganglionic cells with axons exhibiting thickening attributed to myelin. ***H***, A drawing of the human vagus nerve. Images in the public domain reproduced from (Key, 1876). ***I***, Von Kölliker illustrated a ganglion cell from the jugular ganglion of the vagus nerve of a dog, showing the origin of a nerve fiber and its division into a finer branch (c) and a thicker branch (p). Image in the public domain reproduced from Von Kölliker (1896) according to US Copyright Act (Title 17 of the United States Code).

During the late eighteenth and early nineteenth centuries, anatomists began to describe microscopic structures in the nervous system, often referred to as “globules” (Chvatal, 2015; Mathis et al., 2025). Among the earliest contributors, Christian Ehrenberg (1795–1876) distinguished between nerve fibers and ganglia, describing nerves as composed of cylindrical tubes and ganglia as containing more complex, varicose structures (Ehrenberg, 1836). He also examined and drew the human vagus nerve as a bundle of thick fibers (Fig. 3*A*). Although it remains unclear whether he examined vagal ganglia specifically, his observations marked an early step toward distinguishing neuronal elements.

Theodor Schwann (1810–1882) similarly described nerve fibers as tubular structures containing a “white substance,” consistent with myelin (Fig. 3*B*; Schwann, 1839). While he extensively documented ganglion cells in other systems, vagal ganglia were not a primary focus of his work. As a side note, Schwann, together with Matthias Jakob Schleiden (1804–1881), a German botanist, is generally regarded as a principal founder of cell theory.

One of the earliest direct observations of vagal sensory neurons was provided by Adolph Hannover (1814–1894). In 1844, using chromic acid preparations and optical refinements to enhance visualization, he described ovoid cells in fish ganglia with processes resembling those of pseudo-unipolar neurons (Fig. 3*C*; Hannover, 1844). His work demonstrated an emerging understanding that nerve fibers arise from cell bodies.

Friedrich Bidder (1810–1894) extended these observations (Bidder, 1847), describing vagal neurons in both fish and calf with prominent axons and suggesting continuity between nerve fibers and ganglion cells (Fig. 3*D*). His work may represent one of the earliest depictions of mammalian vagal sensory neurons (Fig. 3*E*).

Later, Axel Key (1832–1901) and Gustaf Retzius (1842–1919) used osmium tetroxide to visualize myelinated fibers and described bipolar ganglion cells in fish (Key, 1876). Their samples, obtained from pike and treated with osmium tetroxide, were reported to reveal the myelin sheath (Fig. 3*F*,*G*). The human vagus nerve is also depicted as a bundle of varicose fibers (Fig. 3*H*).

Despite these advances, early preparations provided limited detail, and fundamental questions remained unresolved, including the nature of connections between fibers and cell bodies and the number of processes extending from individual neurons. These studies reflect a transitional period in which the cellular organization of the nervous system was beginning to emerge but remained incompletely understood.

## The “Black Reaction” and the Cellular Organization of Vagal Sensory Neurons

The development of the Golgi staining method by Camillo Golgi (1843–1926) in 1873 marked a turning point in the study of vagal sensory neurons (DeFelipe, 2025). This technique, based on the precipitation of silver chromate, randomly stains a small subset of neurons in their entirety, rendering them in striking black detail against a clear background—hence the term “black reaction” (DeFelipe, 2025). By selectively labeling individual neurons in their entirety, this technique enabled detailed visualization of neuronal morphology and played a central role in establishing the neuron doctrine.

Early applications of Golgi-based methods confirmed that vagal sensory neurons share key morphological features with other sensory ganglia, including characteristic T- or Y-shaped processes. Albert von Kölliker (1817–1905) described such neurons in the jugular ganglion, noting their bifurcating processes and association with satellite cells (Fig. 3*I*).

**Figure 4.**
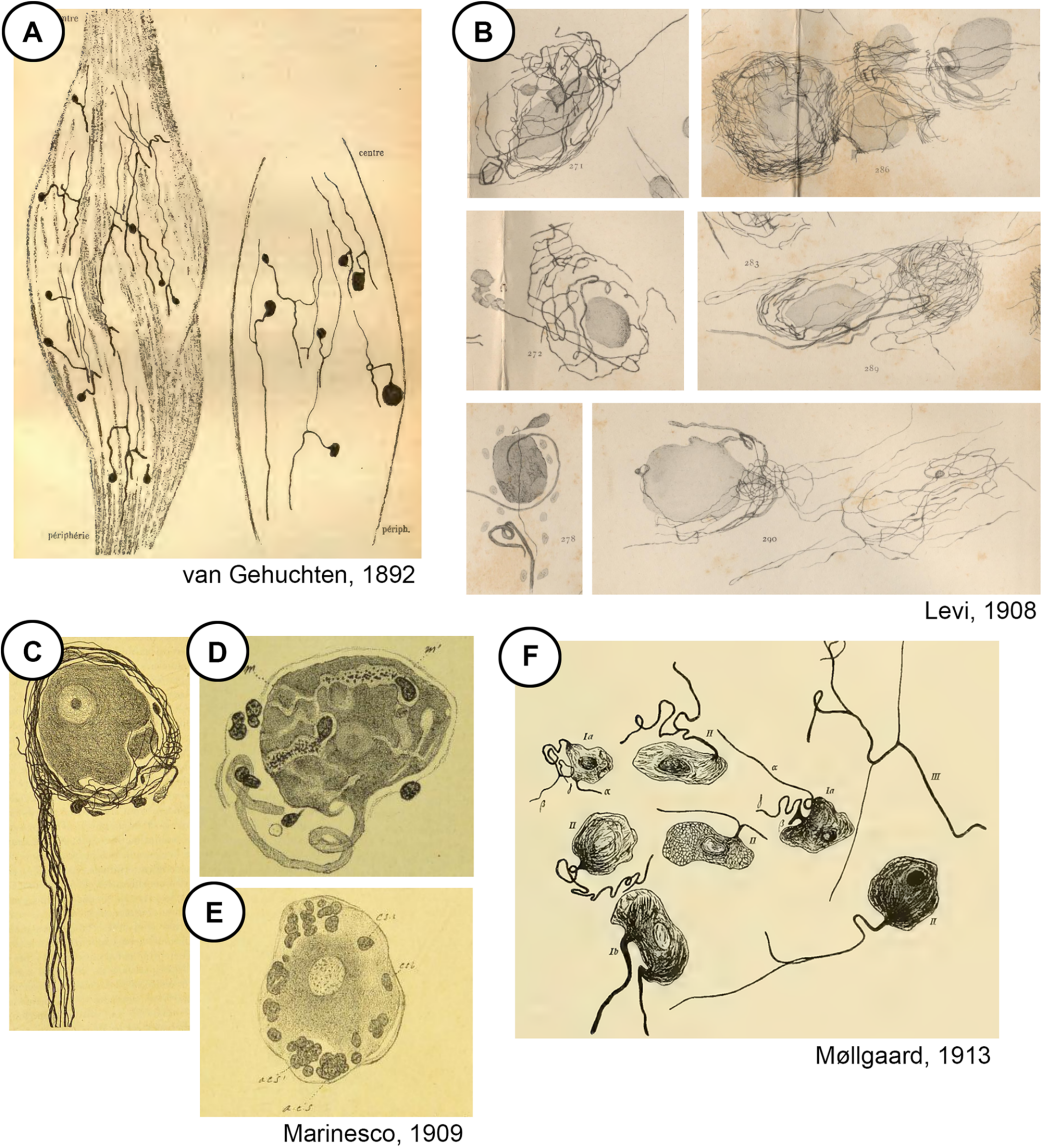
Representative examples of silver-stained vagal sensory neurons from early investigators. ***A***, Drawings of Golgi-stained neurons in the nodose ganglion obtained from a newborn dog (left) and cat (right). Image in the public domain reproduced from Van Gehuchten (1892). ***B***, Drawings by Levi of representative nodose ganglion neurons stained using the Golgi method and obtained from middle-aged men. Image in the public domain reproduced from Levi (1908). ***C–E***, Marinesco reported numerous observations of injured vagal sensory neurons stained using the Cajal method. In ***C***, a neuron in the dog nodose ganglion is shown 18 d after a 3 s compression of the vagus nerve. In ***D***, grafted vagal sensory neurons in the dog spleen are depicted; the dark cells are interpreted as macrophages (m) forming tunnels into the neuron. In ***E***, acetic acid was injected into the dog nodose ganglion to induce proliferation of cells in the neuronal capsule. Images in the public domain reproduced from Marinesco (1909) according to US Copyright Act (Title 17 of the United States Code). ***F***, Møllgaard illustrated nerve cells and axons of the cat nodose ganglion from preparations stained with silver according to the Cajal method. Image in the public domain reproduced from Møllgaard (1913).

Subsequent investigators expanded these observations across species. Arthur Van Gehuchten (1861–1914) demonstrated similar morphologies in multiple mammals (Fig. 4*A*; Van Gehuchten, 1892), while Giuseppe Levi (1872–1965) provided detailed comparative descriptions and morphometric analyses (Fig. 4*B*; Levi, 1908). Georges Marinesco (1863–1938) extended the approach to experimental pathology, documenting morphological changes following nerve injury and chemical insults (Fig. 4*C*; Marinesco, 1909). Notably, he also reported striking observations in which transplanted vagal ganglia (e.g., grafted into the spleen of another dog) exhibited macrophages appearing to form “tunnels” within neurons (Fig. 4*D*). Marinesco sought to illustrate what he termed “neuronophagia,” the process by which nerve cells are engulfed and destroyed by phagocytic cells. In another model, he injected acid acetic into the nodose. He also observed the proliferation of satellite cells around neurons of chemically injured neurons (Fig. 4*E*). Lastly, Holger Møllgaard (1885–1973) examined the effects of vagal nerve section and extirpation, correlating morphological changes in neurons with alterations in respiratory function. His preparations of the cat nodose ganglion are particularly striking (Fig. 4*F*).

**Figure 5.**
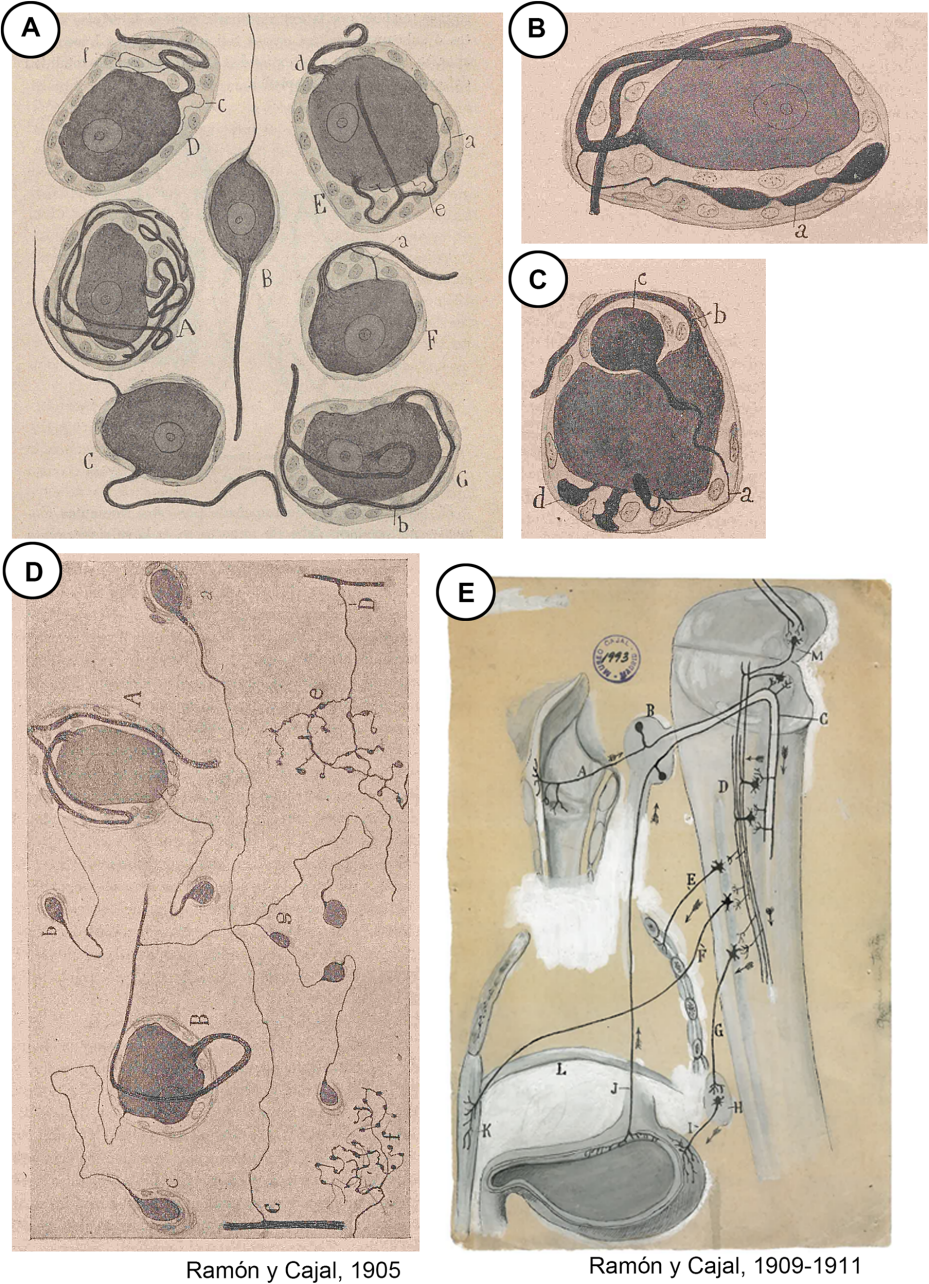
Contribution of Cajal to the study of normal vagal sensory neurons. ***A***, Representative bipolar neurons from the human nodose ganglion of the fenestrated and glomerular types by Cajal. ***B***, Vagal sensory neuron from a donkey with an axon decorated by a chain of beads. ***C***, Neuron with dendrites and a large terminal. ***D***, From young man, neurons with enlarged terminals at distance from the cell body. All images are in the public domain and reproduced from Cajal (1905) according to US Copyright Act (Title 17 of the United States Code). ***E***, Vagal reflexes from Figure 312 of Cajal volume one of his famous “Histologie” (Cajal, 1909–1911).

Among these contributions, the work of Santiago Ramón y Cajal (1852–1934) stands out for its clarity and conceptual depth. Using refined Golgi methods (sometimes called Cajal stain), Cajal produced detailed descriptions of vagal sensory neurons, identifying pseudo-unipolar organization and cataloguing a wide range of morphological variations (Cajal, 1899–1904; Cajal, 1905). Not only Cajal masterized the Golgi method but introduced relevant modifications that were fundamental to offer more systematic and best results (Merchán et al., 2016). He classified a wide range of neuronal types within the nodose ganglion, including bipolar cells, neurons with glomerular configurations, and fenestrated forms (Fig. 5*A*). He also identified neurons with various forms of pouch-like structures (“bolsa”; Fig. 5*B*–*D*). At the time, these atypical morphologies seen in various parts of the peripheral nervous system were the subject of intense debate, with investigators questioning whether they represented pathological states, artifacts of preparation, or normal biological variation (de Castro, 2016).

Finally, Cajal extended his observations beyond morphology to propose functional circuit diagrams of vagal pathways (Fig. 5*E*). One particularly notable illustration depicts individual vagal afferents with their peripheral terminals in visceral organs (such as the stomach and pharynx) and central projections to the medulla and spinal cord (Cajal, 1899–1904; Cajal, 1909–1911). Directional arrows emphasize the sensory nature of these pathways and their integration into motor reflexes to peripheral organs. His accompanying text discusses reflex functions such as coughing and vomiting—interpretations that remain fundamentally consistent with current understanding.

**Figure 6.**
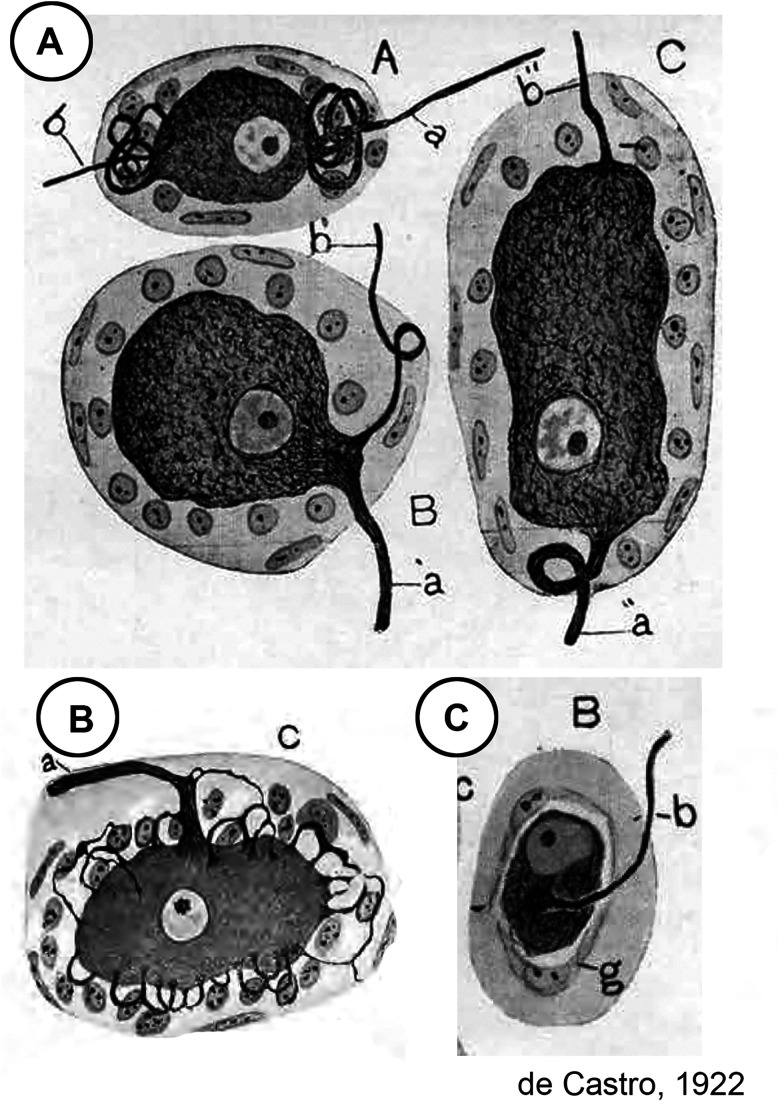
Contribution of de Castro to the study of normal vagal sensory neurons. ***A***, Various types of bipolar neurons in the human nodose ganglion, with their crown of satellite cells. ***B***, Vagal sensory neuron of the fenestrated type from a normal human nodose ganglion. ***C***, Vagal sensory neuron from a human fetus. All images are in the public domain and reproduced from de Castro (1922b) according to US Copyright Act (Title 17 of the United States Code).

One of Cajal's student, Fernando de Castro (1896–1967), further advanced the field through systematic and quantitative analyses (de Castro, 1921; de Castro, 1922b). He confirmed the diversity of neuronal morphologies within vagal ganglia and described distinct subtypes based on soma shape, axonal structure, and pericellular organization. His detailed classifications, including fenestrated and looped processes, represent one of the most comprehensive morphological accounts of vagal sensory neurons in both normal and pathological conditions. Examples of several of these neuronal types in the normal human nodose ganglion, including typical bipolar cells, are shown in Figure 6*A*–*C*. Finally, de Castro should also be commended for quantifying the relative proportions of different neuronal types and for providing early morphometric analyses. His work is further distinguished by a careful and comprehensive comparison with the existing literature of his time.

As a side note, De Castro also revealed the relationship between vagal innervation and arterial chemoreceptors and baroreceptors (de Castro, 2009). This observation is of particular importance because of its role in elucidating the cardiorespiratory reflexes investigated by Corneille Heymans (1892–1968), work that ultimately led to his award of the 1938 Nobel Prize in Physiology or Medicine. Independently, Oleksandr Chernyakhivsky (1869–1939), a Ukrainian disciple of Cajal, also demonstrated that the aortic bodies are innervated almost exclusively by the nodose ganglion of the vagus nerve (Marco et al., 2022).

**Figure 7.**
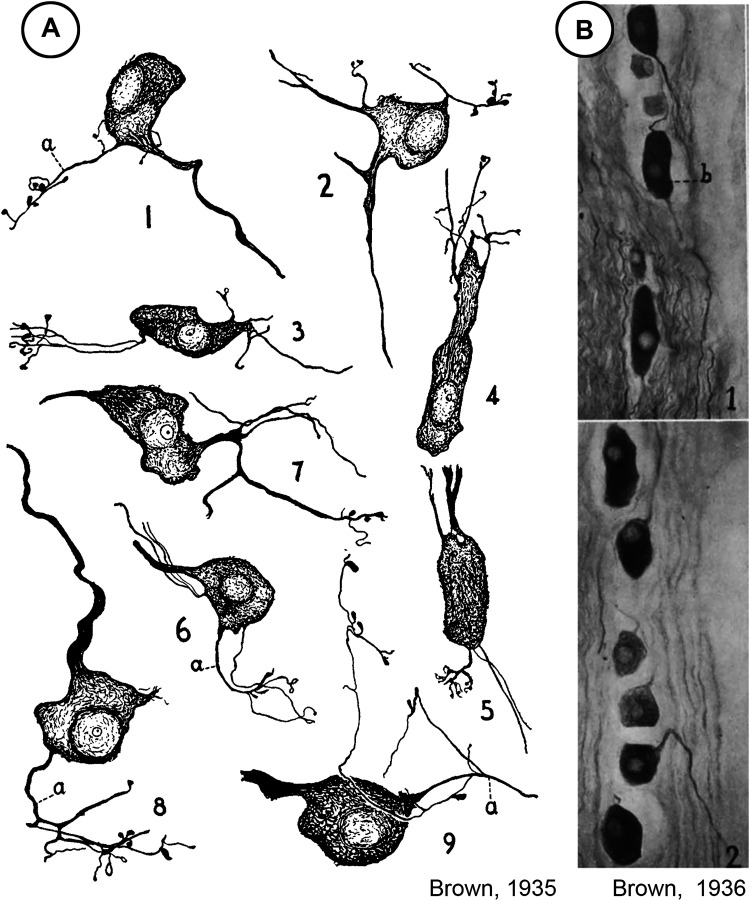
Margaret Brown description of displaced vagal sensory neurons. ***A***, The vagus nerve of newly hatched chicks was stained with the method of Cajal. Various types of bipolar neurons were observed among nerve fibers in a branch of the vague nerve and called “displaced vagal sensory neurons.” Image reproduced from Brown (1935) with permission from John Wiley and Sons (license #6250830613518). ***B***, Photomicrograph of Cajal-stained displaced vagal sensory neurons in the vagus nerve of a puppy. Image reproduced from Brown (1936) with permission from John Wiley and Sons (license #6252561117070).

Later studies continued to refine these observations. Notably, Margaret Brown reported the presence of “displaced” neurons within the vagus nerve trunk of birds and mammals (Brown, 1935; Brown, 1936), suggesting that sensory neurons may not be confined exclusively to canonical ganglia (Fig. 7*A*,*B*).

Overall, the Golgi era established the cellular identity of vagal sensory neurons and revealed their morphological diversity, laying the foundation for subsequent functional and molecular studies.

## Seeing Vagal Fibers and Peripheral Endings

Parallel to the study of neuronal cell bodies, investigators sought to characterize the fiber composition of the vagus nerve and its peripheral terminations. Early histological studies focused on questions such as the relative proportions of myelinated and unmyelinated fibers and the contributions of sensory, motor, and sympathetic components.

**Figure 8.**
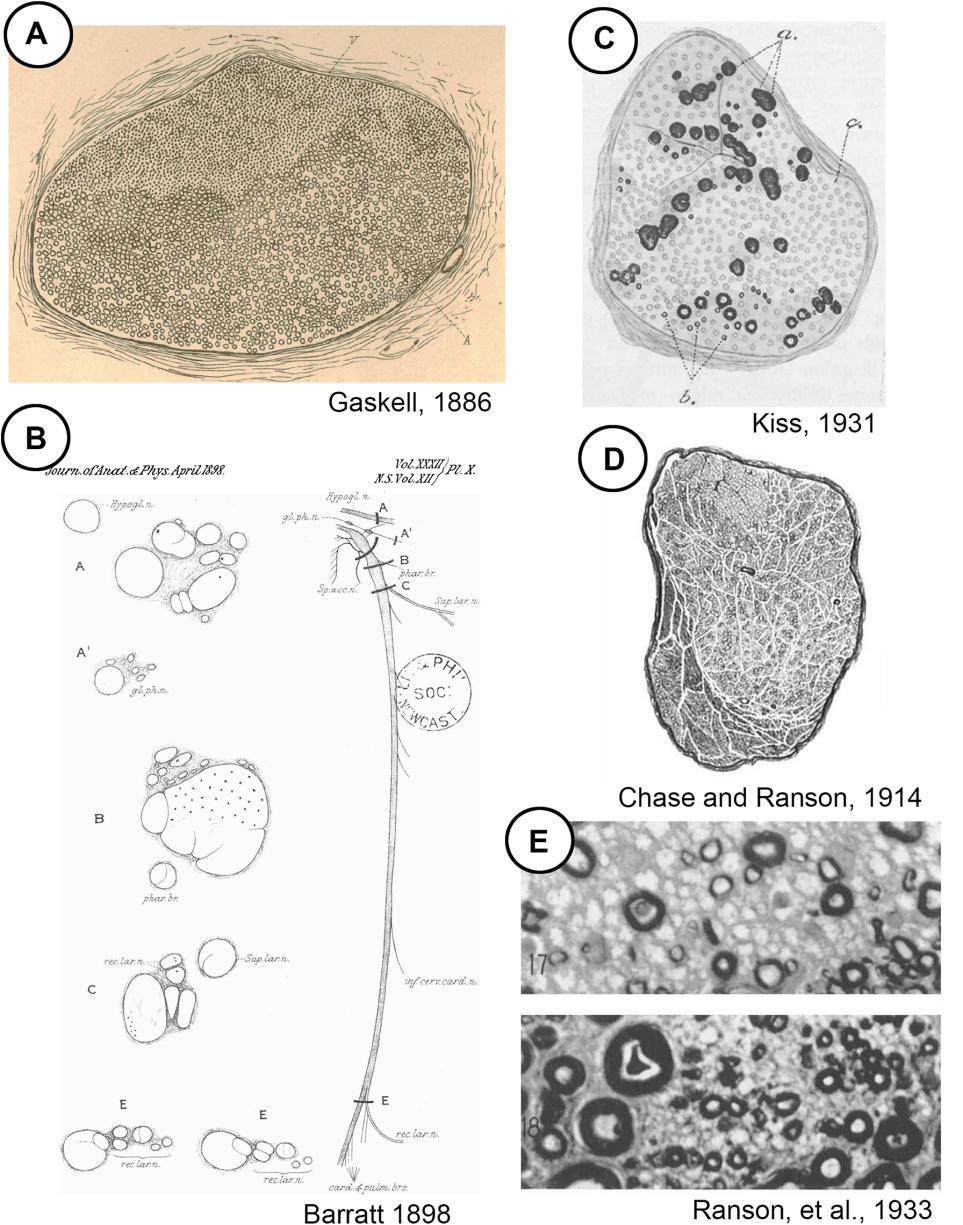
Early microscopy of the fibers composition of the vagus nerve. ***A***, Sections of the dog vagus nerve were stained with osmic acid to highlight different types of fibers. This section is in the spinal accessory nerve attached to the jugular ganglion. Image in the public domain reproduced from Gaskell (1886). ***B***, Barratt produced an early analysis of the composition of the human vagus nerve using sections stained with aniline blue. Image in the public domain reproduced from Barratt (1898). ***C***, Kiss used the Weigert method to stain the vagus, here at the cervical level in a man. Myelinated fibers are seen in thick black outline and interpreted by him as sympathetic fibers. Image reproduced from Kiss (1931). ***D***, Chase and Ranson examined the dog vagus nerve in detail. Section of pyridine silver-stained vagus nerve from a dog at the level of the subclavian artery. Image in the public domain and reproduced from Chase and Ranson (1914). ***E***, Ranson and colleagues examined with great care the composition of the cat vagus nerve on sections stained with osmic acid. Images are from the junction between the nodose and jugular ganglia (top) and from the accessory nerve before its junction with the vagus (bottom). Image reproduced from Ranson et al. (1933) with permission from John Wiley and Sons (license #6253670757036).

Walter Gaskell (1847–1914) provided one of the earliest systematic analyses, demonstrating that unmyelinated fibers predominate in distal segments of the vagus nerve (Fig. 8*A*). His words remain strikingly relevant today: “Again, an examination of the vagus nerves at their entrance into the diaphragm shows that they are here composed almost entirely of non-medullated fibres; so overwhelming indeed is the proportion of ‘non-medullated to medullated’ (referring to their myelinated status) in this part of the nerve that no doubt whatever can exist but that the motor nerves of the stomach and intestines must be included among these non-medullated fibres” (Gaskell, 1886). His analysis was based on the dog vagus nerve using a classic histological technique (osmic acid) that stains the myelin sheath of peripheral nerve fibers black (Gaskell, 1886). Subsequent studies by John Wakelin Barratt (1862–1956) confirmed these findings in humans (Barratt, 1898; Fig. 8*B*).

At the time, there was also ongoing controversy regarding the presence of sympathetic fibers within the vagus nerve. For example, Ferenc Kiss (1889–1966) argued that the vagus contained a large proportion of thin myelinated fibers of sympathetic origin (Kiss, 1931). Although he produced numerous detailed illustrations (Fig. 8*C*), his conclusions were later challenged. In particular, Stephen Walter Ranson (1880–1942) and colleagues demonstrated the presence of unmyelinated vagal fibers rather than sympathetic fibers in the mammalian vagus nerve (Chase and Ranson, 1914; Ranson et al., 1933; Fig. 8*D*,*E*).

However, early fiber counts using histological dyes were often variable. More accurate fiber counts became possible with the advent of electron microscopy. In 1967, Friede and Samorajski examined the mouse vagus nerve at the ultrastructural level (Friede and Samorajski, 1967), followed by numerous studies across different animal species (Gabella and Pease, 1973; Mei et al., 1980; Prechtl and Powley, 1990). Today, this line of research continues in humans (Safi et al., 2016; Biscola et al., 2024).

**Figure 9.**
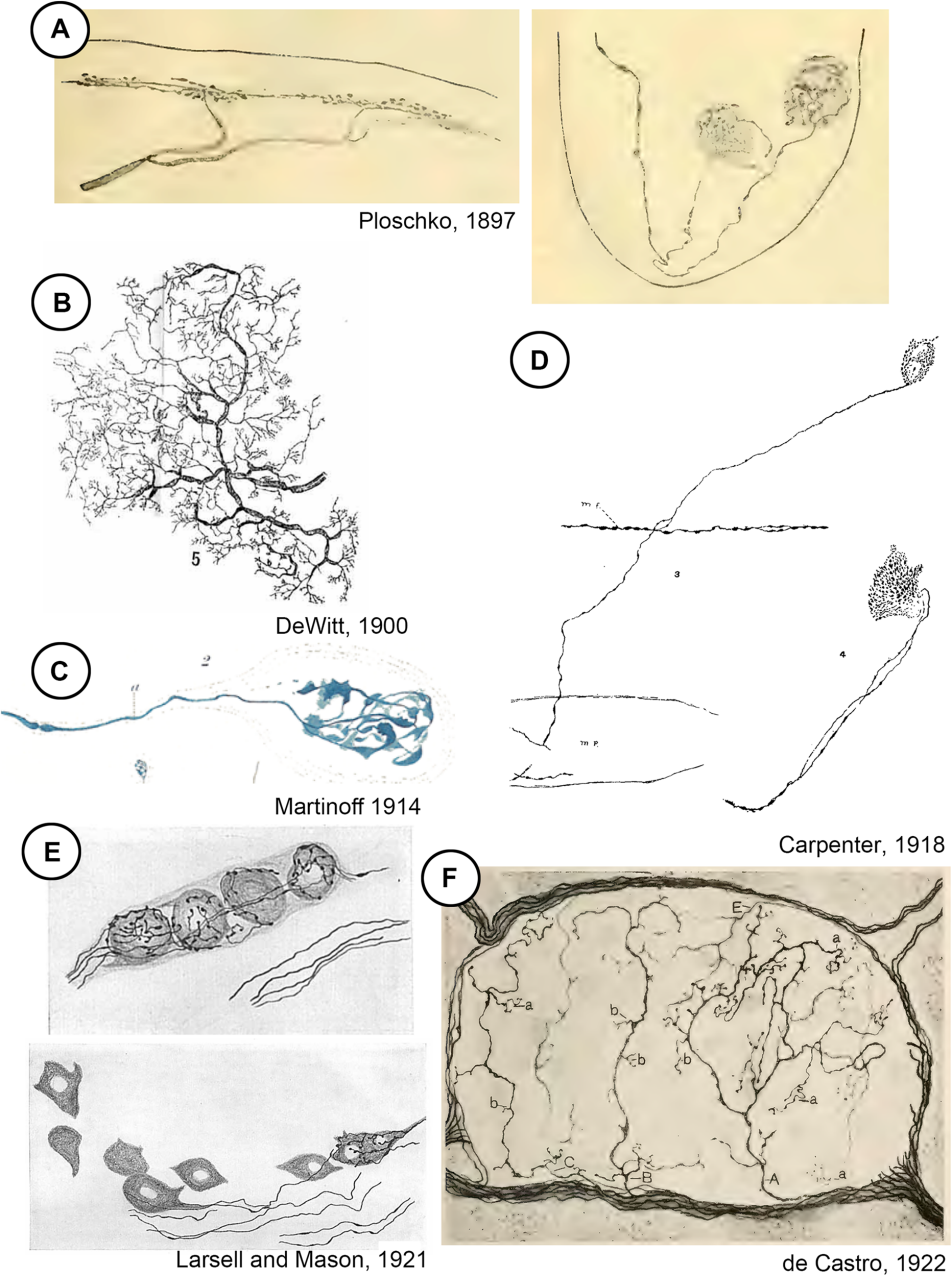
Early studies of specialized nerve endings in stained organs. ***A***, Ploschko illustrated a variety of nerve endings in tissues stained with methylene blue, taken from respiratory organs. The example on the left is from the epiglottis of a rat and indicates subepithelial terminal arborization. The example on the right is from the trachea of a dog and shows a varicose nerve fiber terminating in specialized endings around a ganglion. Image in the public domain and reproduced from Ploschko (1897). ***B***, DeWitt examined the esophagus of a young cat using sections treated with methylene blue and fixed in ammonium molybdate. As a notable example, in the mucosa she observed the stained terminal branching of a large, myelinated sensory nerve, with telodendria extending into the epithelium. Image in the public domain and reproduced from DeWitt (1900). ***C***, Methylene blue-stained myelinated nerve fiber ending in the human pericardium. Image in the public domain and reproduced from Martynoff (1914). ***D***, Carpenter described examples of “end tufts” from the longitudinal muscularis of the small intestine of a dog. Note how the so-called end tufts closely resemble what are now known as intraganglionic nerve endings. Image in the public domain and reproduced from Carpenter (1918). ***E***, Larsell analyzed rabbit lung innervation using methylene blue and described fibers terminating in ganglia. Following a unilateral nerve cut, he observed ganglia that appeared either normally innervated (top) or largely lacking innervation (bottom). Image in the public domain and reproduced from (Larsell and Mason, 1921). ***F***, de Castro (1922a) described richly innervated pancreatic islets in the rat using a Cajal stain, producing highly detailed images. Image in the public domain and reproduced from de Castro (1922a) according to US Copyright Act (Title 17 of the United States Code).

Although fiber composition was increasingly well characterized, the visualization of peripheral endings remained challenging for a long period of time. Nonetheless, techniques such as methylene blue staining enabled partial visualization of terminal arborizations in visceral organs. As early as 1897, Adam Ploschko, a little known histologist from Russia, described the innervation of the respiratory organs and categorized different types of terminal endings, which he termed “Endbäumchen” (“little trees”; Ploschko, 1897). Examples of these structures can be seen in Figure 9*A*, based on tissues obtained from dogs and rats. Morphologically, individual fibers are clearly visible, with features reminiscent of specialized vagal sensory endings (Ploschko, 1897).

Later investigators including, among other examples, Lydia Maria DeWitt (1859–1928; DeWitt, 1900), a little known pathologist by the name of Martynov (Martynoff, 1914), Frederick Walton Carpenter (1876–1925; Carpenter, 1918), and Olof Larsell (1886–1964; Larsell and Mason, 1921) described diverse terminal structures in the respiratory and gastrointestinal systems, often suggesting sensory functions based on morphology and location. Representative images of these authors are available in Figure 9*B*–*E*. Interestingly, Larsell and colleagues attempted to determine the origin of the fibers they observed in the lungs by sectioning the vagus nerve unilaterally and comparing the two sides within the same animal (Fig. 9*E*). On this basis, some fibers were interpreted as motor and others as sensory of mixed, vagal, and sympathetic origins.

Both Cajal and de Castro produced refined image of the fibers within visceral organs. However, once again, one must reference de Castro as a master illustrator. For instance, he dedicated entire work on the innervation of the pancreas (de Castro, 1922a). In it, one can see bundles and individual fibers around ducts, vessels, and the islets of Langerhans (Fig. 9*F*). He makes numerous seminal observations including that the pancreas is innervated by separate nerve supplies to acini, islets of Langerhans, and blood vessels. He also showed that myelinated fibers mainly serve ganglia and vessels of the pancreas, while unmyelinated fibers (perhaps including vagal sensory) form terminal networks into islets of Langerhans.

The above studies were limited by the inability to definitively trace fibers back to their vagal origins. This limitation persisted until the development of anterograde tracing techniques in the late twentieth century (Clerc and Condamin, 1987; Neuhuber, 1987), which allowed direct mapping of projections from vagal sensory neurons to peripheral targets. Subsequent advances, including viral tracing and genetically encoded reporters, have further refined our understanding of vagal innervation (Borgmann et al., 2021; Lowenstein et al., 2023; Cao et al., 2024; Fox and Serlin, 2024).

## Pathological Alterations of Vagal Sensory Neurons

Interest in pathological changes of the vagus nerve dates to the nineteenth century, when gross anatomical alterations were associated with disease states. Among examples, Charles Fernet (1838–1919) described a “larger and firmer” vagus nerve with a “grayish-pink, hydrangea-like” appearance in a 58-year-old woman who died of pneumonia and pleurisy (Fernet, 1878).

**Figure 10.**
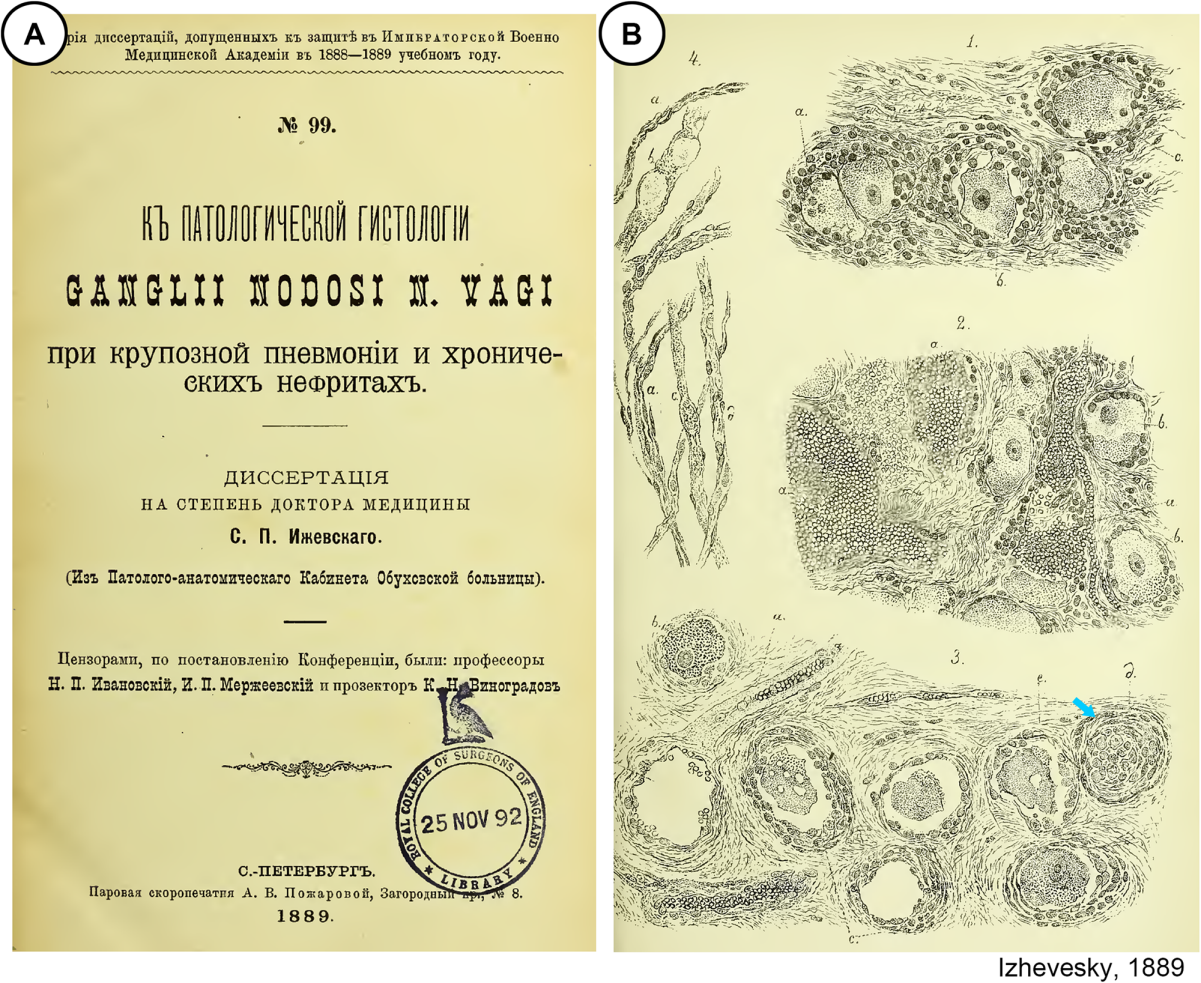
Izhevesky's dissertation on the diseased human nodose ganglion. ***A***, Front page of Izhevesky's medical dissertation in Russian. Its original title was translated to: “On the pathological histology of the ganglia of the vagus nerve (Ganglii nodosi n. vagi) in cases of lobar pneumonia and chronic nephritis.” ***B***, Main plate of illustration of the diseased nodose ganglion from humans who have died of tuberculosis and/or nephritis. Shrinking neurons, proliferating capsules, and hemorrhages are prominent. Blue arrow indicates a cluster of non-neuronal cells arguably reminiscent of what is now known as Nageotte nodule. All images are in the public domain and reproduced from Izhevesky (1889) according to US Copyright Act (Title 17 of the United States Code).

By the late nineteenth century, attention shifted to microscopic pathology. An early and notable contribution is the work of Sergey Izhevesky (Izhevesky, 1889), who examined nodose ganglia from patients with pneumonia and chronic nephritis for his medical dissertation (Fig. 10*A*). He described hemorrhages, inflammatory infiltrates, and degenerative changes in neuronal cell bodies and fibers of the nodose ganglion (Fig. 10*B*). Tissue staining was performed using picrocarmine, hematoxylin, Grenacher's carmine, and Ehrlich's reagent. Although limited by the absence of control samples, his work represents one of the earliest studies of vagal neuropathology.

**Figure 11.**
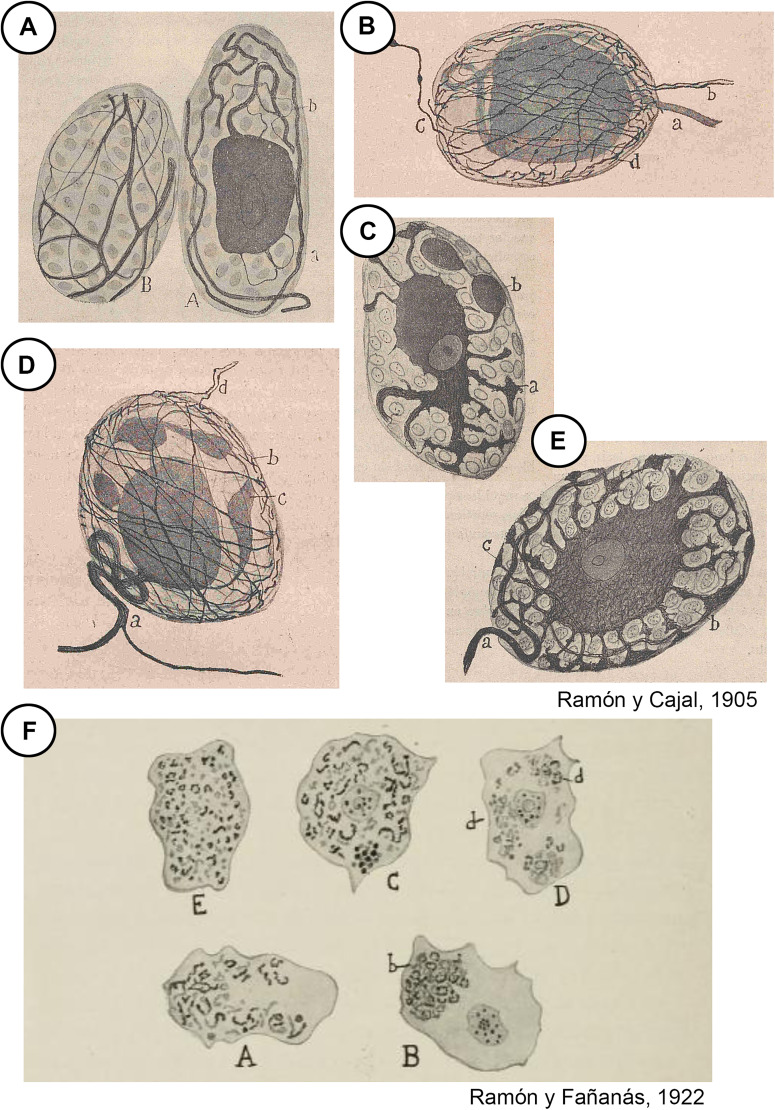
Contribution of Cajal to the study of diseased vagal sensory neurons. ***A***, Fenestrated cells with fine loops in the nodose ganglion of an elderly man (60 years). ***B***, Neuron with an unusual pericellular arborization. ***C***, A senescent ganglion cell of a man of 60 years old. The cell shows newly formed appendage and large terminal spheres. ***D***, Vagal sensory neurons with enlarged terminals. ***E***, Example of a cell suffering from what Cajal called “senile irritation.” All images are in the public domain and reproduced from Cajal (1905). ***F***, In a dog that died of rabies, Ramón y Fañanás described nodose ganglion neurons with a rugged outline and accumulation of abnormal reticulum arrange din loops, granules, or rings. Image in the public domain reproduced from Ramón y Fañanás (1922) according to US Copyright Act (Title 17 of the United States Code).

Cajal also examined pathological and age-related changes, describing morphological alterations in elderly individuals, including irregular cell shapes and increased cellular complexity (Cajal, 1905). According to him, ganglia in humans over 70 years old show anomalies such as more frequent fenestrated cells (Fig. 11*A*,*B*,*D*). He also noted the presence of new cell type known as “celula desgarrada” or “thorned cell” (Fig. 11*C*,*E*). This is a cell with rugged outlines and infiltration of satellite cells and sometimes complicated tangle of filaments or extremely enlarged protrusions. He described such a cell as suffering from “senile irritation.” Ramón Fañanás (1885–1936), who was Cajal' son (and scientific disciple), extended these observations to infectious disease, documenting changes in rabies (Ramón y Fañanás, 1922). One can see fragmented inclusions and granular materials accumulating in sensory neurons of the dog nodose ganglion (Fig. 11*F*).

**Figure 12.**
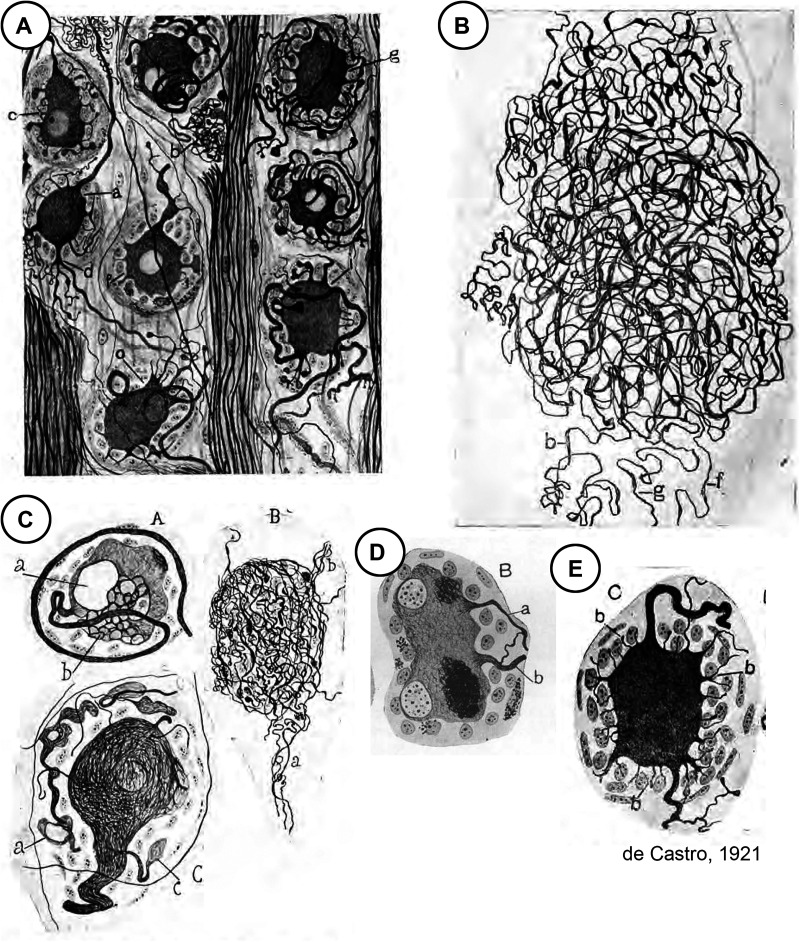
Contribution of de Castro to the study of diseased vagal sensory neurons. ***A***, Nodose ganglion with numerous processes, thick endings, short expansions crowned with knobs, with fibrous loops, torn with signs of irritation, etc. Anomalies according to de Castro represent regenerative figures following in man, brought about by section of the peripheral root during a surgical intervention (observed at 6 days postsurgery). ***B***, Newly formed fibers from the nodose that accumulated after the vagal section. ***C***, Nodose ganglion abnormal-looking neurons from an alcoholic with a cell containing vacuoles (letter A), a cell with accumulation of neurofibrils (letter B), and a cell innervated by swollen neurofibrils. ***D***, A neuron with two nuclei and dark cytoplasmic inclusion form the nodose ganglion of a patient who died with pellagra. ***E***, Vagal sensory neuron of a child (8 years old) with pulmonary tuberculosis, with many processes radiating from the cell body. All images are in the public domain and originally from de Castro (1921) according to US Copyright Act (Title 17 of the United States Code).

Fernando de Castro provided the most comprehensive analysis of pathological conditions, examining ganglia from patients with a wide range of diseases (de Castro, 1921; de Castro, 1922b). He impressively lists having examined the ganglia of individuals who have died of cancer, vertebral tuberculosis, alcoholism, syphilis, paralysis, amyotrophic lateral sclerosis, osteomalacia, beriberi, old age, tetanus, intoxications, leishmaniasis, pellagra, and rabies. His observations were not restricted to vagal neurons but encompassed neurons located in various sympathetic and sensory ganglia (de Castro, 2016). In the nodose ganglion, he described both degenerative and regenerative changes, including axonal swelling, vacuolization, and abnormal pericellular structures (Fig. 12*A*–*E*). His observations of the nodose ganglia from alcoholics are particularly striking. He noticed neurons with large vacuoles and accumulation of fibrils (Fig. 12*C*). As another example, de Castro described a cell taken from a pellagra patient with its nuclei at the extremes of the soma, peripheral, and containing numerous argentophilic degenerative granules (Fig. 12*D*). Many of the medical conditions listed above are now known causes of injury to the peripheral nervous system (Elafros et al., 2022). Overall, de Castro's work anticipated modern concepts of neuropathy and demonstrated that vagal sensory neurons are affected in diverse pathological contexts.

Subsequent studies incorporated electron microscopy of the human vagus nerve, revealing ultrastructural alterations in conditions such as tuberculosis and diabetes (Jabonero, 1957; Guy et al., 1984). More recent research has extended these observations to molecular and cellular levels, further refining our understanding of peripheral neuropathies (Sapio et al., 2016; Shiers et al., 2025). However, molecular studies dedicated to the pathologies of the human vagus nerve remain sparse.

## Conclusion: A Continuing Neuroanatomical Journey

The study of the vagus nerve has progressed through successive methodological eras, from gross anatomy to histology, electron microscopy, and modern molecular approaches. Despite these advances, many central questions have persisted, including the functional specialization of vagal pathways, their organization, and their role in disease.

Historical analysis reveals recurring patterns, including the early recognition of laterality, neuronal diversity, and pathological alterations. While some aspects of classical research—such as comparative anatomy and detailed morphological description—have become less prominent, modern techniques have introduced unprecedented specificity (Ottaviani and Macefield, 2022).

Recent studies indicate substantial heterogeneity among vagal sensory neurons, with multiple subtypes defined by molecular and functional characteristics. Importantly, this diversity was already anticipated by early investigators such as Cajal and de Castro, who recognized morphological variation within vagal ganglia.

Thus, contemporary research does not replace earlier work but extends it, providing a more detailed resolution of questions that have long shaped the field. Understanding this historical continuity offers valuable perspective on current advances and highlights enduring challenges in vagal neurobiology.

## Synthesis

Reviewing Editor: Arvind Kumar, KTH Royal Institute of Technology Decisions are customarily a result of the Reviewing Editor and the peer reviewers coming together and discussing their recommendations until a consensus is reached. When revisions are invited, a fact-based synthesis statement explaining their decision and outlining what is needed to prepare a revision will be listed below. The following reviewer(s) agreed to reveal their identity: NONE.

I apologize for the delay. It took some time to secure a reviewer for this manuscript. In fact I had to give up the hope of getting another reviewer after more than 10 declines.

In any case this is an important account of history and the reviewer has recognized this. They have made a few suggestions to further improve the manuscript and embellish the historical perspective.

Here are the comments from the reviewer. I look forward to reading the revision.

## Author Response

Synthesis of Reviews:

Synthesis Statement for Author (Required):

I apologize for the delay. It took some time to secure a reviewer for this manuscript. In fact I had to give up the hope of getting another reviewer after more than 10 declines.

In any case this is an important account of history and the reviewer has recognized this. They have made a few suggestions to further improve the manuscript and embellish the historical perspective.

Answer: Thank you for your efforts. The wait was worth it since the feedback from the reviewers is excellent. Please find our revised manuscript.

Here are the comments from the reviewer. I look forward to reading the revision.

This historical perspective on the evolution of the research on the structure and terminals of vagal neurons (from the ganglion, to the peripheral structures) is very interesting and overall can be considered a relevant piece of work to understand how these evolved, the pivotal moments, scientists and techniques that were capital for unraveling the subject. However, there are some issues that deserve to be corrected to consider this work as publishable in a prestigious journal like eNeuro:

1-Abstract: It may include the names/facts of at least the most relevant contributors to the subject.

Answer: We agree with the suggestion. Please see our revised abstract.

2-The work by Willis (lines 54-55) should be cited before in that page, when chronologically corresponds (i.e.: before Ludwig´s work).

Answer: We agree and made the requested move.

3-When the contribution of Theodore Schwann is cited (line 77) it would be orientative for the reader to detail that Schwann was, together with Schleiden, the main contributors to the "Cell Theory" in the 1830s.

Answer: Thank you. This information which I was unaware of was added. "As a side note, Schwann, together with Matthias Jakob Schleiden (1804-1881), a German botanist, is generally regarded as a principal founder of cell theory." 4-Line 99: Regarding the Golgi method, it was described in 1873 and deserves to include a bibliographic citation.

Answer: we included the date of 1873 and, if acceptable, repeated the reference of "DeFelipe, 2025" which describes quite well the historical context.

5-Line 108: Rüdolf Albert von Koelliker is more known as "Albert von Koelliker". Please, use both names or Albert.

Answer: we made the requested change.

6-Line 117: Regarding the contribution of Marinesco´s, it would be great to know which is the current interpretation of the cited sentence "macrophages appearing to form "tunnels" within neurons".

Answer: Good question. Marinesco sought to illustrate what he termed "neuronophagia," the process by which nerve cells are engulfed and destroyed by phagocytic cells.

7-Santiago Ramón y Cajal is more frequently referred as "Cajal, S.R." in citation and reference lists. I strongly suggest to adopt this all along the text.

Answer: We made the requested changes.

8-Line 124: Cajal not only masterized the Golgi method but introduced relevant modifications that were fundamental to offer more systematic and best results. Please, add this information to the main text and eventually include a reference supporting this (either the original paper or the technical book: Cajal &De Castro, 1933).

Answer: We made the requested changes.

9-Line 136: Cajal indeed include similar descriptions in the original "Textura..." (1899-1904), which was later translated into French for its publications, with moderate additions and changes, as "Histologie...".

Answer: We now cite the "textura".

10-Line 140: Cajal has a number of important direct scientific disciples (for a recent work in the subject, see: De Castro, 2019 Front. Cell. Neurosci.). In this sense, Fernando de Castro should be cited as "one of the..." or "one of the more relevant..." or "one of the last direct disciples...".

Answer: Answer: We made the requested changes.

11-There are cited two papers of de Castro that were originally published in 1922: please, add "1922a" and "1922b" in the text and reference list to properly identify to which of them the main text refers.

Answer: we differentiated 1922a and 1922b.

Also regarding De Castro in the subject of the current manuscript, it should be discussed the relationship or arterial chemoreceptors and baroreceptors (De Castro, 1926 Trav. Lab. Invest. Biol. Univ. Madrid; De Castro, 1928 Trav. Lav. Invest. Biol. Univ. Madrid) to vagal innervation with a modern perspective (Ros &de Castro, 2020 Anat. Rec.; de castro &López-Barneo, 2026 Acta Physiol. Scand.) because of the relevance of this in unravelling the cardio-respiratory reflexes by Heymans and his Nobel Prize 1938. They were vey cited also the chapter in Penfield´s treaty (de Castro, 1932), and maybe deserve citation, here.

Answer: Thank you. This information which I was unaware of was added.

12-Line 164: the current reader maybe need an explanation on "medullated and non-medullated fibres" to properly understand that in modern terminology.

Answer: good point. We clarified the terminology.

13-Gaskell´s work (lines 168-169) deserve a citation after "using a classic histological technique that stains the myelin 169 sheath of peripheral nerve fibers black", as well as the clarification on which classid histological technique he obtained such results.

Answer: I added the missing information: osmic acid.

14-Maybe the most delicate observation is about the sentence in line 178: "However, early histological investigations were often variable and prone to artifacts". The reader must take home a clear summary of the authors´ that published still valid observations, distinguishing from the cited artifacts. Either this is explicitly summarised in the Conclusions or please consider a Table commenting all the details included in the present work.

Answer: we agree that our statement was too strong. We changed for "However, early fiber counts using histological dyes were often variable." 15-Lines 234-236: Jorge Ramón Fañanás was son (and scientific disciple) in this order, more than the written one. He signed "Ramón Fañanás".

Answer: yes, it is changed accordingly.

16-Maybe the author/s did not notice a relatively recent publication in which the work by the Ukranian (then Soviet) disciple of Cajal, Oleksandr Chernyakhivsky, performed in different fields, including the innervation of the heart: it deserve to be included in the current work (Marco et al., Neurosciences and History 2022; 10(2): 46-60 -I´ve checked and with this info, is freely accesible in internet-).

Answer: thank you for this suggestion. The article from Marco is indeed highly interesting and cited in the section regarding the carotid body innervation.

17-Finally, in different figure legends of the extremely nice figures, the author/s write "All images are in the public domain": Can be more explicit about the origin of this, please? Answer: to the best of our knowledge this refers to U.S. Copyright Act (Title 17 of the United States Code), stating that images published before 1931 are in public domain.
